# Diagnosis of tuberculosis with autoimmune hepatitis–systemic lupus erythematosus overlap syndrome: a case report

**DOI:** 10.1186/s13256-022-03572-8

**Published:** 2022-11-05

**Authors:** Shekhar, Nikita Yogesh Gupta, Avi Ravi Harisingani

**Affiliations:** Grant Government Medical college and Sir JJ group of hospitals, JJ Marg, Mumbai, Maharashtra 400008 India

**Keywords:** Tuberculosis, Systemic lupus erythematosus, SLE, TB, Autoimmune hepatitis, AIH, Lupus

## Abstract

**Background:**

There is ample evidence indicating that immunosuppressive therapy or immune dysregulation in systemic lupus erythematosus increases the risk for tuberculosis. Interestingly, a few case reports suggest that tuberculosis could also be a risk factor for systemic lupus erythematosus and other autoimmune diseases.

**Case presentation:**

We report the case of a 32-year-old Indian patient who was co-diagnosed with tuberculosis, systemic lupus erythematosus, and autoimmune hepatitis without any history of prior immunosuppression. This stresses the complex relationship between tuberculosis and autoimmune diseases.

**Conclusion:**

Further research is warranted in this field to unfold the complex relationship between tuberculosis and systemic lupus erythematosus. It is essential to establish clear guidelines for the management of coexisting tuberculosis and systemic lupus erythematosus to promote individualized treatment.

## Background

Systemic lupus erythematosus (SLE) is an autoimmune disease that displays varied presentations and can involve multiple organ systems. Having a prevalence of 14–60 cases per 100,000, the burden of SLE in India is comparatively low [1]. Tuberculosis (TB) is an infectious disease that can involve multiple organ systems, having varied presentations, and can thus often be confused with SLE. India has one of the highest numbers of TB cases worldwide, making it vital to understand all the intricacies of TB [2].

While there is abundant evidence to show that TB, more so extrapulmonary, can occur as a complication of SLE due to immunosuppressive therapy or immune dysregulation, there have also been a few reported cases revealing concomitant diagnoses of TB and SLE as well as antecedent diagnosis of TB prior to SLE. There have been similar rare case reports involving other autoimmune diseases as well. This leads us to speculate that TB may be a risk factor for SLE as well as other autoimmune diseases. The current case report briefly explores this complex relationship between TB and SLE.

Moreover, there are no treatment guidelines established for cases of concomitant TB and SLE, which highlights the importance of reporting such cases, especially in countries where they are endemic [3, 4].

## Case report

A 32-year-old Indian female presented with continuous fever for 5 days, recorded as 100–101 °F by the patient, associated with chills in the evening and relieved by over-the-counter antipyretics. She complained of a dull aching pain in the lower abdomen, without any specific exacerbating/relieving factors. Subsequently, she started to experience right upper quadrant abdominal pain, lesser in severity as compared with the lower abdominal pain. This was followed by insidious-in-onset and gradually progressive yellowish discoloration of the eyes, dark-colored urine, abdominal distention, and nonbilious, nonprojectile vomiting episodes. Fatigue was noted over the past 1 month. She denied burning micturition, abnormal discharge per vaginum, rash, or bleeding tendencies.

The patient reported an episode of jaundice in her adolescence. She denied any addictions and had no history of significant blood loss, IV drug abuse, or tattooing. Her bowel and bladder habits were unaltered.

On examination, pulse was 120 beats/min and regular, with all peripheral pulses palpable. Blood pressure in the right arm in supine position, respiratory rate, and oxygen saturation were recorded as 110/70 mmHg, 20 cycles/min, and 98%, respectively. Pallor, icterus, and bilateral pitting pedal edema were present. Clubbing and peripheral lymphadenopathy were absent. On palpation and percussion, tenderness in the right hypochondrium and hypogastrium and shifting dullness was present. The patient was first admitted under surgical care to rule out acute cholangitis.

Laboratory testing revealed:

**Table:** 

Investigation	Patient value	Reference range
Hemoglobin	10.4 g/dL	11–15 g/dL
Leukocyte count	40,000/mm^3^	4.0–11.0 × 10^3^/mm^3^
Neutrophils	90%	40–60%
Platelet count	379,000/mm^3^	100–400 × 10^3^/mm^3^
Total bilirubin	10.1 mg/dL	0.2–0.8 mg/dL
Direct bilirubin	5.1 mg/dL	0.0–0.3 mg/dL
Aspartate transaminase (AST)	72 IU/L	8–48 IU/L
Alanine transaminase (ALT)	40.6 IU/L	7–55 IU/L
Alkaline phosphatase (ALP)	89.8 IU/L	45–117 IU/L
INR	1.60	<1.2
Serum albumin	2.3 g/dL	3.5–5.0 g/dL

**Fig. 1:**
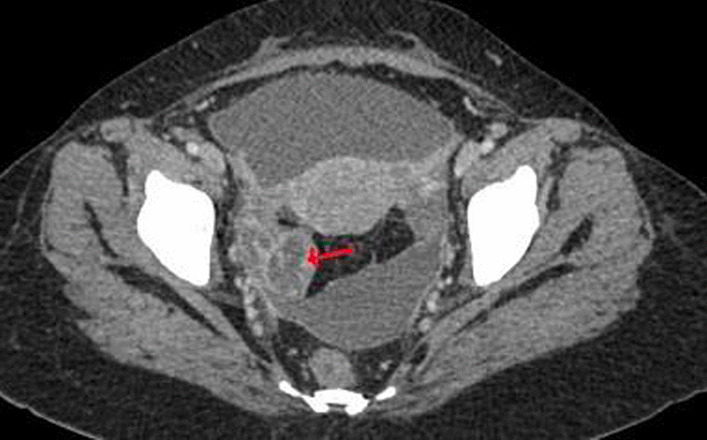
CECT transverse cut pelvis showing peripherally enhancing septate pelvic collection (red arrow)

**Fig. 2:**
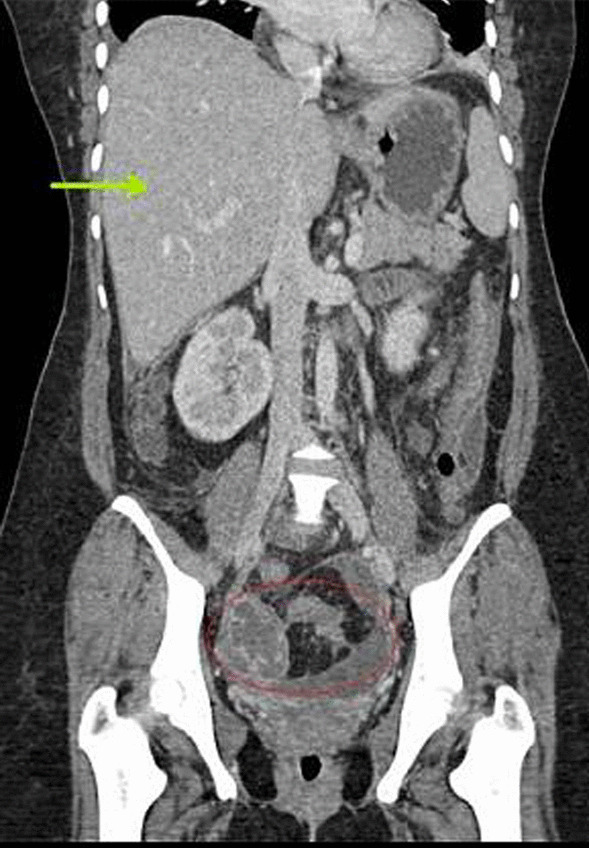
CECT coronal cut showing hepatomegaly (green arrow) and pelvic collection (red circle)

**Fig. 3:**
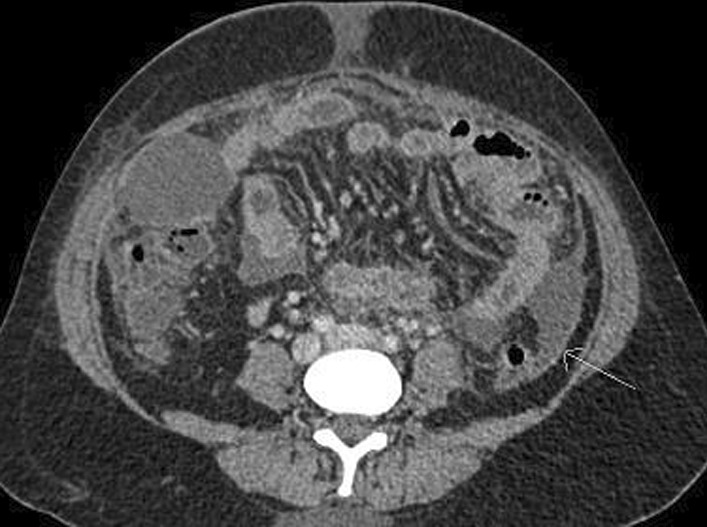
CECT transverse cut showing peritoneal enhancement (white arrow)

Contrast-enhanced computed tomography (CECT) scan of the abdomen and pelvis revealed a normal biliary tree, moderate hepatomegaly with homogeneous enhancement pattern and no focal lesions; ascites and diffuse peritoneal thickening, large loculated peripherally enhancing pelvic collection (approximately 100 cc, measuring 4.8 × 5.5 × 3.9 cm^3^). The collection had incomplete septa and was seen extending laterally on both sides from the pouch of Douglas, closing abutting bilateral ovaries (Figs. 1, 2, 3).

Evaluation of icterus was further sought; the patient tested negative for hepatitis B surface antigen (HBsAg), hepatitis A and E IgM antibodies, hepatitis C antibodies, and HIV enzyme-linked immunosorbent assay (ELISA) antibody. Erythrocyte sedimentation rate (ESR) was 40 mm in the first hour, and C-reactive protein (CRP) level was 1.2 mg/dL. Considering the liver enzyme levels, the patient was not tested further for other viral etiologies of hepatitis. Tuberculous hepatitis was also ruled out as imaging did not reveal any suggestive findings such as macronodules/micronodules/focal lesions [5].

Unexplained deranged liver function tests (LFT) in a young female with anemia, without any history of blood loss, raised ESR and CRP levels, with a previous history of jaundice, raised high suspicion for autoimmune pathology.

Autoimmune workup was done, which revealed:

**Table:** 

ANA by IFA	Positive (1:100) with cytoplasmic filament pattern
Anti-Ku	114, +++
Anti-PCNA	17, +
Anti-smooth muscle antibody	Positive
LKM1 antibody	Negative
p-ANCA	Negative
c-ANCA	Negative
Antiphospholipid antibodies (IgG, IgM, IgA)	Negative
C3 complement	179.0 mg/dL (90–180 mg/dL)
C4 complement	20.0 mg/dL (10–40 mg/dL)
Total IgG	2510 mg/dL (700–1600 mg/dL)
LDH	476 IU/L (106–211 IU/L)
Direct Coombs	Positive
Indirect Coombs	Negative
Peripheral smear	Normochromic normocytic picture (no schistocytes seen)

*Anti PCNA* antibodies to proliferating cell nuclear antigen,* LKM1 antibody* Liver Kidney Microsomal Antibody,* pANCA* Perinuclear anti-neutrophil cytoplasmic antibodies,* cANCA* Cytoplasmic anti-neutrophil cytoplasmic antibodies,* IgG* Immunoglobulin G,* IgM* Immunoglobulin M,* IgA* Immunoglobulin A

Urinalysis and urine microscopic examination showed:

**Table:** 

Investigation	Patient value	Reference values
Appearance	Hazy	Clear/slightly hazy
Blood	Strongly positive	Absent
Albumin	+++	Absent
Protein/creatinine ratio	2.05 g/day	<0.2 g/day
RBC	40–50/hpf	0–5/hpf
Pus cells	10–12/hpf	0–6/hpf
Epithelial cells	8–10/hpf	Absent
Granular casts	Few	Absent

Renal biopsy was performed and demonstrated lupus nephritis class V with index of activity and index of chronicity of 0/24 and 0/12, respectively. The patient fulfilled the European League against Rheumatism (EULAR)/American College of Rheumatology (ACR) 2019 criteria for diagnosis of SLE with a score of 14 (fever: +2; autoimmune hemolysis: +4; renal biopsy showing lupus nephritis class V: +8). The high positive anti-smooth muscle cell antibody titres, normal complement levels, and raised IgG levels, with negative viral markers, drug and alcohol history, indicated the diagnosis of autoimmune hepatitis (score of 6 on the simplified scoring system and 17 on the revised international AIH group scoring system). We reached a probable diagnosis of AIH–SLE overlap syndrome.

To evaluate the cause of gross ascites and tubo-ovarian mass revealed on imaging, ascitic fluid analysis was performed. It revealed:

**Table:** 

Test	Result
RBC count	None
Total WBC count	1500/mm^3^
Neutrophil	1400/mm^3^
ADA	76.24 U/L
Protein	6 g/dl
Sugar	72 mg/dl
Serum ascitic albumin gradient (SAAG)	0.48 g/dl

*RBC* red blood cell,* WBC* white blood cell,* ADA* adenosine deaminase

Ascitic fluid adenosine deaminase (ADA) levels were checked as ADA levels >36I U/L suggest tuberculosis [6]. Among tumor markers, CA-125 was elevated at 152.2 U/mL, whereas carcinoembryonic antigen, alpha-fetoprotein, and beta-hCG levels were within reference values. Fitz–Hugh–Curtis syndrome and malignancy were ruled out based on the imaging findings and tumor marker levels, respectively. In view of CECT abdomen and pelvis findings along with large loculated pelvic collection and bilateral tubo-ovarian mass, SAAG of 0.48, and high ascitic fluid ADA levels, in an endemic setting, we concluded a diagnosis of tuberculous pelvic inflammatory disease with tuberculosis peritonitis. *Mycobacterium tuberculosis* was not detected by ascitic fluid cartridge-based nucleic acid amplification test (CB-NAAT). The patient was advised to undergo ultrasound-guided liver biopsy (for histopathological examination for grading and staging AIH) and laparoscopic omental and peritoneal biopsy (for histopathological examination, CB-NAAT, and mycobacterial growth indicator tube test), but the patient did not consent to the procedure.

## Treatment

Due to the existing liver disease, drugs least likely to cause hepatocellular damage (streptomycin + levofloxacin + ethambutol) were administered to treat the tuberculous PID and peritonitis. Over the next 2 weeks, her liver function normalized, so we switched the above regimen to first-line agents for tuberculosis: isoniazid, pyrazinamide, and rifampicin; and continued them for 6 months [7]. Standard weight-based dosing was followed for all the drugs [8].

An ACE inhibitor was added for management of proteinuria due to lupus nephritis, taking into consideration the current guidelines for class V and index of activity 0/24 (nephrology recommendations were including regarding the same). Steroid/immunosuppressive regimens were not administered in tandem with the recent management guidelines for AIH and lupus nephritis, current tuberculosis status, and her improving liver function tests.

## Follow-up

We closely followed the patient to monitor liver function tests, proteinuria, or worsening symptoms. Over 2–3 weeks, WBC counts normalized, hemoglobin showed a rising trend, proteinuria improved (repeat urine protein creatinine ratio of 0.116 compared with 2.05 on admission), and urine microscopy showed disappearance of granular casts, RBCs, and WBCs. Repeat radiological imaging showed that the ascites, peritoneal enhancement, and pelvic collection had resolved.

## Discussion

Infections can trigger autoimmune responses owing to many mechanisms, including molecular mimicry, which evades the T-cell tolerance and causes polyclonal activation of B cells. Type 1 interferon plays a vital role in the pathogenesis of SLE. Excess type 1 interferon causes activation of immature dendritic cells, leading to amplification of autoreactive B and T lymphocytes and apoptotic cells. Presence of high levels of type 1 interferon and lupus antibodies in TB patients strengthens this correlation [3, 9]. Furthermore, antibodies formed against *Mycobacterium* have shown cross-reactivity with DNA [10]. Another possibility is immune dysfunction increasing the susceptibility to both tuberculosis and immune-mediated diseases. However, whether this causes activation of latent tuberculosis or increases the risk of TB remains uncertain [4].

These immunological speculations have been strengthened by studies among SLE and TB patients. Gosh *et al.* conducted a study on a cohort of 70 SLE patients, 20% of whom had antecedent TB. It showed a 40-fold higher prevalence of tuberculosis compared with the regional population (*p* < 0.001, 95% CI) [11]. Ramagopalan *et al.* studied the England dataset to demonstrate a 4.4-fold increase in the incidence of SLE in patients with prior TB infection 1 year after admission and a 2.5-fold rate ratio 5 years after admission (*p* < 0.001, 95% CI). The Oxford record linkage study showed a 5.3 times higher rate of SLE in patients admitted for antecedent TB [4]. These data indicate a relationship between these two entities, although which one precedes the other is difficult to unravel.

SLE causes hyperactivity of the immune mechanism, leading to impaired cellular and humoral immune functions, predisposing to infections such as TB [12]. Various genetic components including deficiencies of complement and mannose-binding lectin, serious disease presentations, and immunosuppressant use have been postulated to explain the predisposition of SLE patients to infections [13]. Infections are responsible for causing up to 50% of morbidity and mortality in SLE patients [13, 14]. This warrants physicians looking out for serious infections such as TB, especially in endemic regions [15]. Furthermore, a point of caution is the similar, nonspecific presenting symptoms of TB and lupus, such as fever, malaise, and weight loss, posing a diagnostic challenge [16, 17]. Several studies have demonstrated lupus nephritis as an independent risk factor for development of TB [3, 18]s. A study conducted at Singapore General Hospital showed a 3.33-fold higher rate of TB in lupus patients as compared with those without SLE [19]. Similarly, Erdozain *et al.* [20] reported a sixfold higher incidence of TB in the SLE group. Studies demonstrate a higher occurrence of extrapulmonary TB as compared with pulmonary TB [16, 18].

Autoimmune hepatitis, previously known as lupoid hepatitis, is liver inflammation of unknown etiology. It predominantly affects women and is associated with hypergammaglobulinemia, autoantibodies, and characteristic histological findings. The presentation of AIH has a wide range, including acute severe to asymptomatic disease [21–23]. AIH is classified into three types based on the autoantibodies detected in the patient. Type I AIH is characterized by anti-smooth muscle antibodies (ASMA) and accounts for 80% of all AIH cases [24]. ASMA, seldomly found in SLE patients, is highly indicative of AIH and hepatic involvement. Although SLE hepatitis is well known, it is rare, leading us to suspect AIH [25, 26].

According to the revised International Autoimmune Hepatitis Group scoring system, our patient’s pretreatment score is 17 (+2 for female, +2 for ALP/AST <1.5, +2 for IgG 1.5–2 times above normal, +3 for ASMA/ANA >1:80, +3 for negative viral marker, +1 for no drugs, +2 for alcohol < 25 g/day, +2 for other immune diseases), which along with her normal complement levels and fulfillment of the EULAR/ACR criteria for SLE 2019, is highly suggestive of an overlap syndrome. Although a pretreatment score of 15 signifies definitive AIH [27] (95% specificity, 97% sensitivity, and 94% accuracy), we could not perform a liver biopsy to confirm the diagnosis, stage, and grade conclusively due to lack of consent. Though challenging due to their similar clinical and biochemical features, it is essential to differentiate between them, owing to the poor prognosis of AIH as compared with lupus hepatitis, which is benign.

AIH–SLE overlap syndrome rarely progresses to severe outcomes such as cirrhosis requiring liver transplantation, hepatocellular carcinoma, and death seen in AIH patients [28]. Overlap syndrome also requires an individualized approach to treatment as each case is unique [29]. As per the management recommendations for AIH [24] (treatment initiated in patients with aminotransferases ten times the upper limit of normal OR aminotransferases five times the upper limit of normal with gamma globulin levels at least two times the upper limit of normal) and class V lupus nephritis, while considering the tuberculosis status as well as improving LFTs, we decided not to administer steroids/immunosuppression. Individualization of treatment for this patient proved to be wise as her clinical condition and laboratory parameters normalized. On further imaging, there was radiological improvement of her tuberculosis.

## Conclusion

We conclude that TB can be a risk factor for SLE and not only vice versa. A similar relationship may exist between TB and other autoimmune diseases. Since both TB and SLE are diseases with a wide spectrum of manifestations, diagnosis of one should not prevent one from looking for the other, particularly in areas where TB is endemic. Further research is warranted in this field to unfold the complex relationship between TB and SLE. It is essential to establish clear guidelines for the management of coexisting TB and SLE to promote individualized treatment.

## Abbreviations

AIH: Autoimmune hepatitis
ADA: Adenosine deaminase
ANA: Antinuclear antibody
ASMA: Anti smooth muscle antibody
CRP: C reactive protein
ESR: Erythrocyte sedimentation rate
LFT: Liver function tests
PID: Pelvic inflammatory disease
PCNA: Proliferating cell nuclear antigen
SAAG: Serum ascites albumin gradient
SLE: Systemic lupus erythematosus
TB: Tuberculosis
